# Piperlongumine Suppresses Proliferation of Human Oral Squamous Cell Carcinoma through Cell Cycle Arrest, Apoptosis and Senescence

**DOI:** 10.3390/ijms17040616

**Published:** 2016-04-23

**Authors:** San-Yuan Chen, Geng-Hung Liu, Wen-Ying Chao, Chung-Sheng Shi, Ching-Yen Lin, Yun-Ping Lim, Chieh-Hsiang Lu, Peng-Yeh Lai, Hau-Ren Chen, Ying-Ray Lee

**Affiliations:** 1 Department of Chinese Medicine, Ditmanson Medical Foundation Chiayi Christian Hospital, Chiayi 600, Taiwan; cych02157@gmail.com; 2 Department of Life Science, National Chung Cheng University, Chiayi 621, Taiwan; ai90395@gmail.com (G.-H.L.); laiweichien@yahoo.com.tw (P.-Y.-L.); 3 Department of Nursing, Min-Hwei Junior College of Health Care Management, Tainan City 736, Taiwan; april@mail.mhchcm.edu.tw; 4 Graduate Institute of Clinical Medical Sciences, Chang Gung University, Chiayi 613, Taiwan; csshi@mail.cgu.edu.tw; 5 Department of Medical Research, Ditmanson Medical Foundation Chiayi Christian Hospital, Chiayi 600, Taiwan; jouyuan22@gmail.com; 6 Department of Pharmacy, College of Pharmacy, China Medical University, Taichung 404, Taiwan; limyp@mail.cmu.edu.tw; 7 Division of Endocrinology and Metabolism, Department of Internal Medicine, Ditmanson Medical Foundation Chiayi Christian Hospital, Chiayi 600, Taiwan; 02602@cych.org.tw

**Keywords:** piperlongumine, human oral squamous cell carcinoma, cell cycle arrest, apoptosis, senescence

## Abstract

Oral squamous cell carcinoma (OSCC), an aggressive cancer originating in the oral cavity, is one of the leading causes of cancer deaths in males worldwide. This study investigated the antitumor activity and mechanisms of piperlongumine (PL), a natural compound isolated from *Piper longum* L., in human OSCC cells. The effects of PL on cell proliferation, the cell cycle, apoptosis, senescence and reactive oxygen species (ROS) levels in human OSCC cells were investigated. PL effectively inhibited cell growth, caused cell cycle arrest and induced apoptosis and senescence in OSCC cells. Moreover, PL-mediated anti-human OSCC behavior was inhibited by an ROS scavenger *N*-acetyl-l-cysteine (NAC) treatment, suggesting that regulation of ROS was involved in the mechanism of the anticancer activity of PL. These findings suggest that PL suppresses tumor growth by regulating the cell cycle and inducing apoptosis and senescence and is a potential chemotherapy agent for human OSCC cells.

## 1. Introduction

Oral cancer encompasses all malignancies originating in the oral cavity. Oral cancer is the sixth most common cancer worldwide and the third most common cancer in developing countries [1]. In Taiwan, oral cancer is the fourth leading cause of cancer deaths in males [2]. Most oral cancer cases are histologically classified as oral squamous cell carcinoma (OSCC) [3,4]. OSCC is an aggressive cancer, and its occurrence is closely associated with cigarette smoking, alcohol consumption and betel nut chewing [3]. In developed countries, exposure to one, two or all of these factors accounted for more than 75% of all oral cancer cases, and further research indicated that the combined effect is multiplicative rather than additive [5]. In Taiwan and Southeast Asia, betel quid chewing has been strongly associated with oral cancer [3,5]. Betel nut extract has been evidenced to promote cell migration and early invasion mediated through the Src kinase/extracellular signal-regulated kinase 1/2 signaling pathway [6,7]. In addition, betel nut extract may increase DNA mutation in human oral tissues and contribute to early-stage oral carcinogenesis [8]. The incidence rate and severity of OSCC have been gradually increasing over the past 10 years. In 2010, OSCC was the fifth most common malignant cancer in Taiwan, with a mortality rate at 13.31 per 100,000 persons. OSCC cases are typically diagnosed through pathological examination by referring to the guidelines proposed by the World Health Organization, and the overall five-year death rate reached 50% after surgery and radiation treatments, in combination with chemotherapeutic agents [9]. Therefore, a novel and effective treatment modality is urgently required for radiation and chemotherapy-resistant OSCCs.

Piperlongumine (PL) is a melanogenesis inhibitor obtained from *Piper longum* L. (fruits, Piperaceae) through activity-guided extraction and isolation. A cell-based, high-throughput screening approach is used to identify PL, which can selectively kill various transformed cell types while sparing primary normal cells [10]. PL can increase reactive oxygen species (ROS) levels and apoptotic cell death in both cancer cells and normal cells engineered to have a cancer genotype, irrespective of the p53 status, with little effect on either rapidly or slowly dividing primary normal cells [10]. Moreover, PL suppresses tumor growth in established tumor xenografts in mice, including human bladder, breast and lung tumors in nude mice and mouse melanoma in B6 mice. PL induced apoptosis in a caspase-dependent manner. Furthermore, blood vessel formation was suppressed in xenograft tumor mice after PL treatment, indicating an antiangiogenesis mechanism of PL in cancer therapy [10]. Despite the anticancer activity of PL in multiple types of cancers, the effect of PL in human OSCC remains unevaluated. In addition to PL, some chemotherapeutic agents, such as cisplatin and paclitaxel, have been investigated: PL treatment was proved to increase cisplatin antitumor activity in head and neck cancer and to induce synergistic antigrowth of human ovarian cancer cells once in combination with either cisplatin or paclitaxel treatment [11,12].

Aerobic conditions are associated with continuous production of free radicals, particularly ROS, which can function in signal transduction, cancer initiation and progression, and the clearance of pathogens during innate immune responses [13]. Antioxidant defense, which deals with the produced ROS, and an oxidant-antioxidant imbalance resulting in an excessive accumulation of ROS are defined as oxidative stress. Oxidative stress was observed to be higher in cancer cells than in normal cells [14]. Moreover, the activation of a specific oncogene, *H-Ras*, can elevate ROS [15]. ROS can cause DNA alternations, strand breaks and damage, leading to cell death. Cancer cells undergoing chronic oxidative stress induce genomic alternations, thus facilitating the detoxification of ROS to prevent cell death and induce tumorigenesis [10,13,16]. It is now widely accepted that constitutively-elevated levels of cellular oxidative stress and dependence on mitogenic and antiapoptotic ROS signaling in cancer cells represent a specific vulnerability that can be selectively targeted by directly or indirectly acting pro- and anti-oxidants and redox modulators; these modulators are together referred to as redox chemotherapeutics, thus representing a novel class of promising anticancer agents [14]. Normal cells or non-transformed cells show lower basal levels of ROS and express a higher antioxidant capacity to prevent treatments that impair ROS metabolism [14].

The antioxidant capacity has been reported to be low in human OSCC cells [17,18]. Hence, this study aimed to develop a novel therapeutic agent by regulating ROS in betel nut chewing-mediated OSCC cells. We explored the chemotherapeutic effects of PL in human OSCCs and present evidence that PL inhibits the growth of human OSCC cells through cell cycle arrest, senescence and ROS-mediated caspase-dependent apoptosis.

## 2. Results

### 2.1. Piperlongumine Suppresses the Growth of Human Oral Squamous Cell Carcinoma

**Figure 1:**
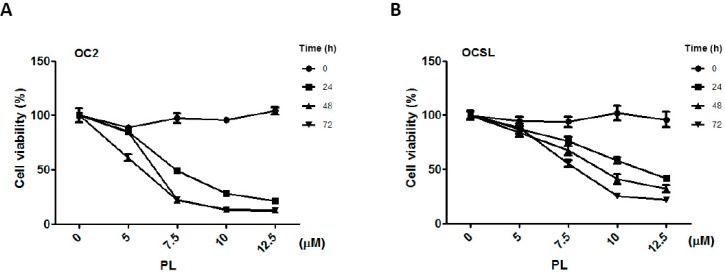
Effect of piperlongumine on the cell viability of human oral squamous cell carcinoma. (**A**) OC2 and (**B**) OCSL cells were incubated without or with various concentrations of piperlongumine, and the cellular viability was measured by the CCK-8 assay. DMSO was used as a negative control. The results are expressed as the mean ± S.D. (*n* = 9) of three independent experiments.

To evaluate the effect of PL on human OSCC cells, the two OSCC cell lines OC2 and OCSL were treated with DMSO (a vehicle) or PL. Cell proliferation after the treatments was investigated using CCK-8 analysis. Figure 1 shows that PL inhibited the proliferation of human OSCC cells in a time- and dosage-dependent manner. The cell morphology was determined through microscopy (Figure S1). The posttreatment IC_50_ of PL in the OC2 cells was 7.4, 5.7 and 6.5 µM at 24, 48 and 72 h, respectively. Moreover, the posttreatment IC_50_ of PL in the OCSL cells was 11.3, 9.2 and 7.0 µM at 24, 48 and 72 h, respectively. Although PL effectively suppressed the growth of both the OC2 and OCSL cells, this result suggests that the OCSL cells were more resistant to the PL treatment (Figure 1).

### 2.2. Piperlongumine Induces G1 Phase Arrest in Human Oral Squamous Cell Carcinoma

**Figure 2:**
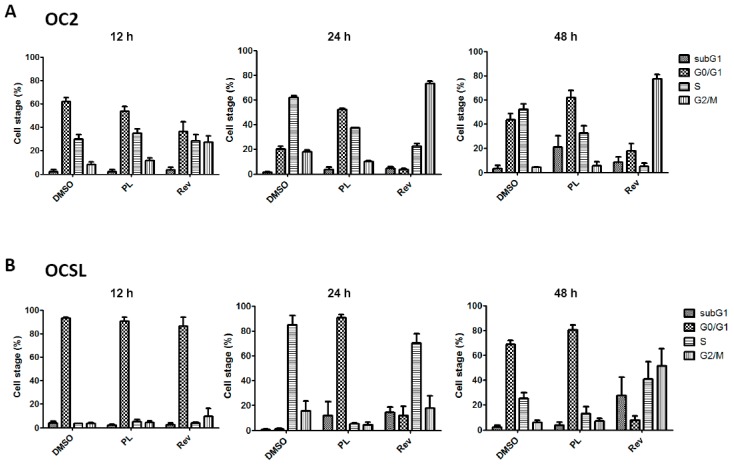
Piperlongumine induces cell cycle arrest at the G0/G1 phase in human oral squamous cell carcinoma. (**A**) OC2 and (**B**) OCSL cells were incubated with DMSO, piperlongumine (10 µM) or reversine (Rev; 10 µM) for 12, 24 and 48 h, and the cell cycle stages were determined by flow cytometric analysis. The data present as the mean ± S.D. of three independent experiments.

**Figure 3:**
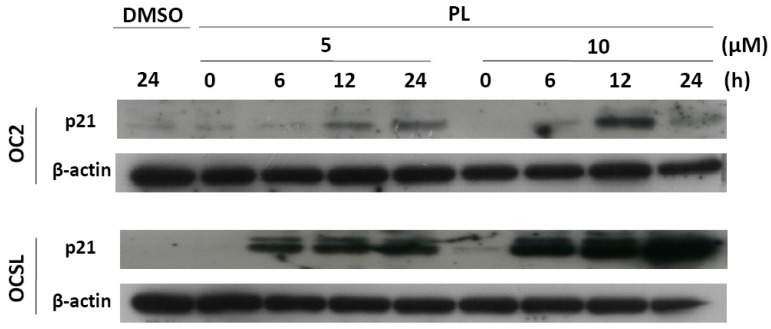
Piperlongumine elevates the expression of p21 in human oral squamous cell carcinoma (OSCC) cells. OC2 and OCSL cells were incubated either with DMSO or with various concentrations of piperlongumine for 0, 6, 12 and 24 h. The expression of p21 was analyzed by Western blotting, and β-actin was used as an internal control.

To determine whether the PL-induced growth inhibition was influenced by cell cycle arrest, OC2 and OCSL cells were incubated with DMSO or PL, and cell cycle was examined through flow cytometry. Reversine was previously used as the positive control for the G2/M phase arrest of cells [19]. Cell cycle arrest at the G0/G1 phase was observed in the PL-treated OC2 and OCSL cells (Figure 2). Moreover, the OCSL cells were more sensitive to PL-induced G0/G1 arrest than were the OC2 cells (Figure 2). A previous study reported p21 and p27 to be cyclin-dependent kinase inhibitors that were involved in response to various stresses, including DNA damage, hypoxia and confluence stress [20]. To confirm the PL-mediated cell cycle arrest in human OSCC cells, p21 expression was examined using Western blotting of PL-treated OC2 and OCSL cells. We observed that PL increased p21 expression in both cell lines in a time- and dosage-dependent manner (Figure 3). The induction level of p21 after PL treatment was higher in the OCSL cells than in the OC2 cells (Figure 3). This observation was consistent with the observation that the OCSL cells were more sensitive to PL-induced G0/G1 arrest than were the OC2 cells (Figure 2).

### 2.3. Senescence Induction in Piperlongumine-Treated Human Oral Squamous Cell Carcinoma

**Figure 4:**
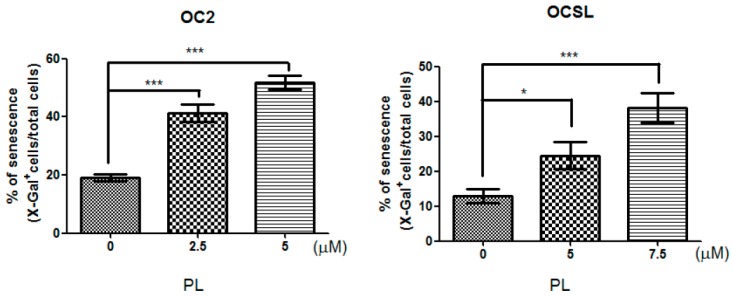
Piperlongumine (PL) treatment induces cellular senescence in human OSCC cells. Senescent cell assays were conducted in OC2 and OCSL cells treated with various concentrations of piperlongumine for 24 h by senescence-associated β-galactosidase (SA-β-Gal) staining, and the percentage of β-Gal staining positive cells was statistically analyzed after staining. Data are presented as the mean ± SD. * *p* < 0.05 and *** *p* < 0.001 as compared to the control (0 μM).

We demonstrated PL-induced p21 overexpression in OC2 and OCSL cells (Figure 3). The biological function of p21 was reported to involve various cellular pathways, including the cell cycle, checkpoints, senescence and terminal differentiation of the cells [21]. Therefore, we further evaluated whether senescence was elevated in PL-treated human OSCC cells. β-galactosidase was used to evaluate PL-induced senescence in the cells. Figure 4 shows the basal level of senescence in OC2 and OCSL cells under DMSO treatment. Senescence was significantly elevated in both OC2 and OCSL cells treated with PL (Figure 4 and Figure S2). However, OC2 cells appeared to be more sensitive to PL-induced senescence than OCSL cells (Figure 4). These data revealed that PL can induce senescence in human OSCC cells.

### 2.4. Piperlongumine Elevates Caspase-Dependent Apoptosis in Human Oral Squamous Cell Carcinoma

**Figure 5:**
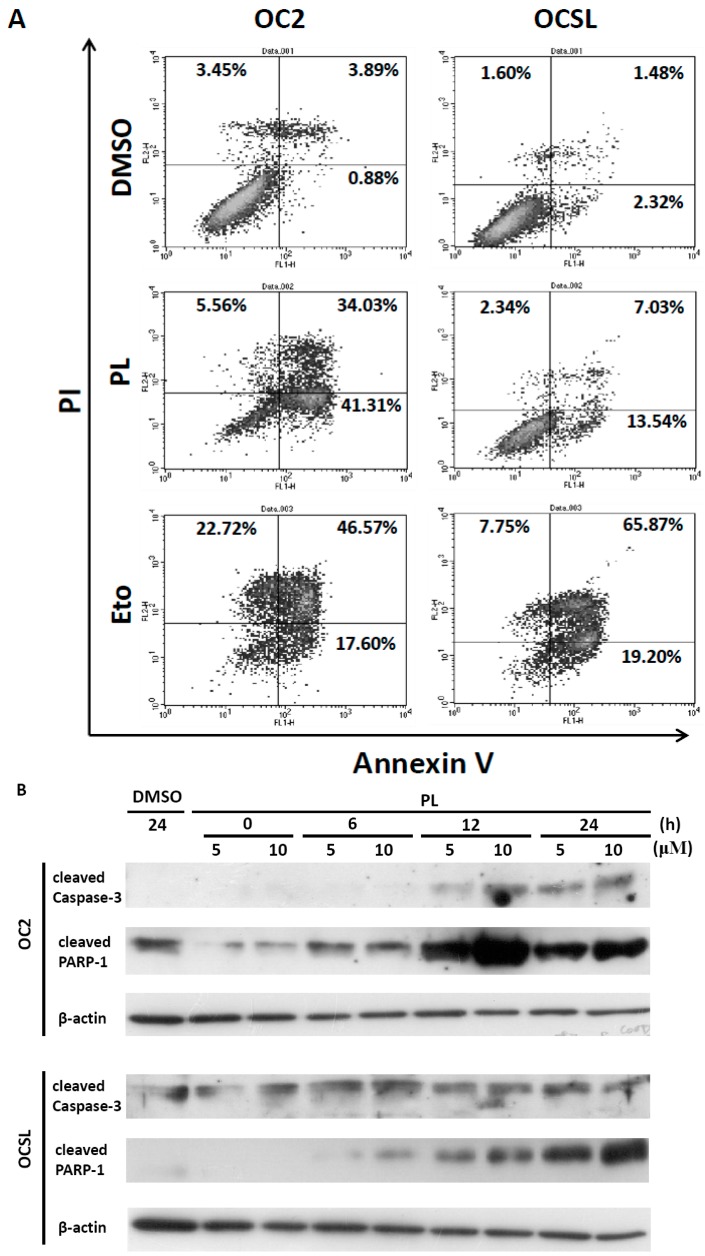

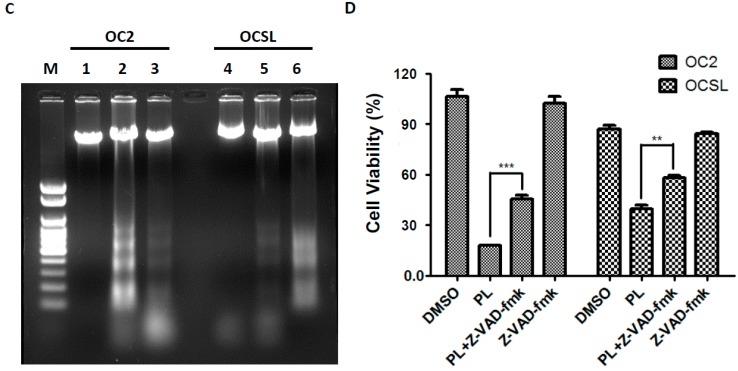
Piperlongumine induces apoptosis in human OSCC cells. (**A**) OC2 and OCSL cells were treated with DMSO, piperlongumine (10 µM) or etoposide (Eto; 50 µM) for 48 h, and apoptotic cells were determined with flow cytometry after PI/annexin-V double staining; (**B**) Cells treated with DMSO or piperlongumine for various times; the expressions of cleavage forms of caspase-3 and PARP-1 were detected by Western blotting; β-actin was used as an internal control; (**C**) DNA fragmentation was examined in the piperlongumine-treated cells for 48 h, and the genomic DNA was extracted and analyzed electrophoretically on 10% agarose gels containing ethidium bromide. M: marker; Lane 1 and 4: DMSO; Lane 2 and 5: PL (10 µM); Lane 3 and 6: reversine (10 µM); (**D**) The cellular viability of OC2 and OCSL cells treated with DMSO, piperlongumine (10 µM), Z-VAD-fmk (a pan-caspase inhibitor; 20 µM) and piperlongumine (10 µM) plus Z-VAD-fmk (20 µM) for 48 h was measured by CCK-8 analysis. The data are presented as the mean ± SD. ** *p* < 0.01; *** *p* < 0.001.

The sub-G1 phase was partially observed in PL-treated OC2 and OCSL cells (Figure 2), suggesting that PL induced the death of the OC2 and OCSL cells. To confirm that apoptosis was induced to reduce the growth of human OSCC cells after PL treatment, OC2 and OCSL cells were treated with DMSO or PL for 48 h; cell apoptosis was then determined through flow cytometry after PI-annexin-V double staining. We observed that PL significantly induced apoptosis in both OC2 and OCSL cells (Figure 5A). Moreover, OC2 cells were clearly more sensitive to PL-mediated apoptosis than were OCSL cells (Figure 5A). This result was consistent with those in Figure 1A,B suggesting that OCSL cells were more resistant to PL treatment. Furthermore, we evaluated the activation of caspase to determine the mechanisms underlying PL-mediated apoptosis in human OSCC cells. OC2 and OCSL cells were incubated with DMSO or PL for various time periods, and the expression of the cleavage forms of caspase-3 and PARP-1 was detected using Western blotting. Figure 5B shows that caspase-3 was activated in the PL-treated OC2 and OCSL cells; PARP-1 was also activated. PARP-1 upregulation was higher in the OC2 cells than in the OCSL cells 12 h after the treatment (Figure 5B). To confirm that apoptosis occurred in the PL-treated cells, DNA fragmentation was examined in the PL-treated OC2 and OCSL cells. DNA fragmentation was strongly observed in the PL-treated OC2 and partially observed in the OCSL cells, respectively (Figure 5C). These data suggested that PL induced apoptosis in human OSCC cells. In addition, we verified that PL-mediated apoptosis played a role in suppressing the growth of human OSCC cells. Figure 5D shows significant growth inhibition of the OC2 and OCSL cells after PL treatment, and this phenomenon was partially reversed by coincubating the cells with Z-VAD-fmk. Altogether, these results suggested that PL provides an anti-human OSCC function by inducing caspase-dependent apoptosis.

### 2.5. Piperlongumine Regulates ROS and Caspase-Dependent Apoptosis in Human Oral Squamous Cell Carcinoma

**Figure 6:**
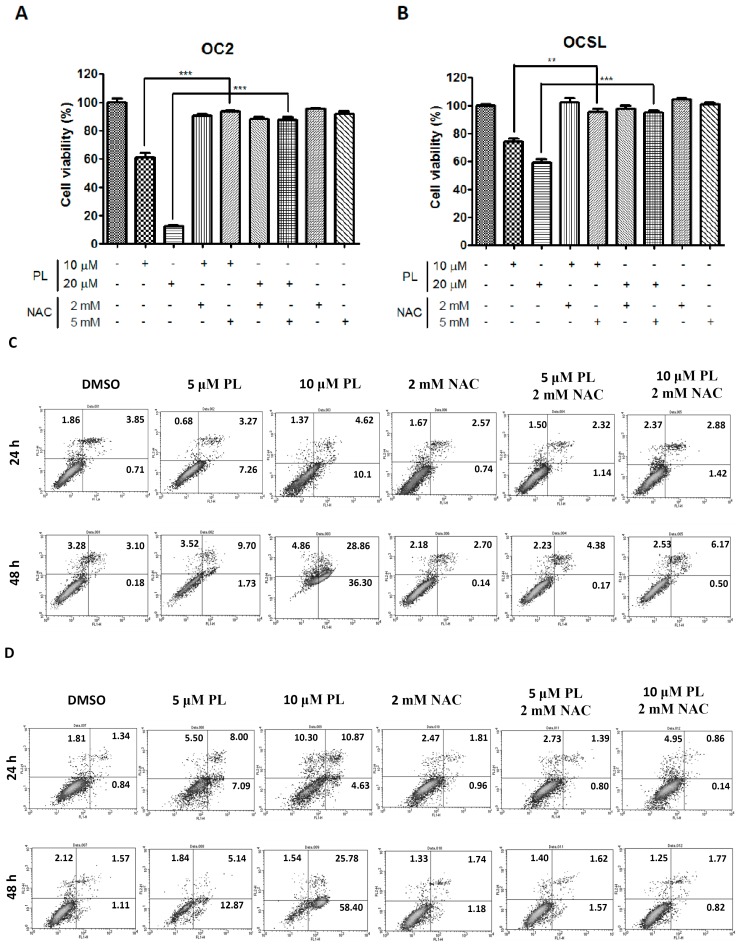

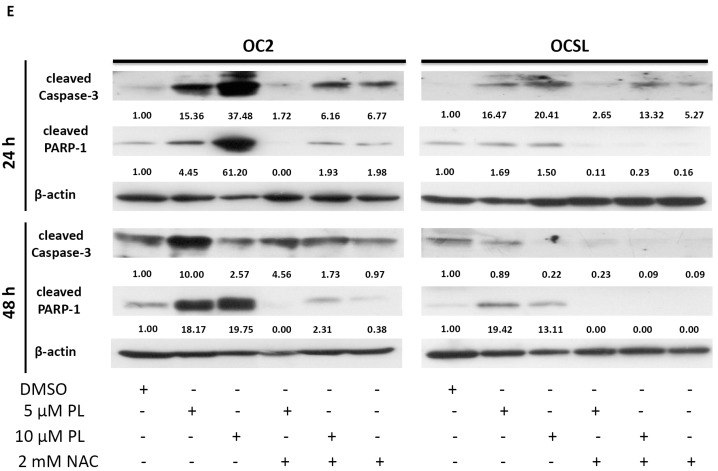
ROS are involved in the piperlongumine-mediated caspase-dependent apoptosis in human OSCC cells. The cell viability of (**A**) OC2 and (**B**) OCSL cells treated with piperlongumine in the presence or absence of *N*-acetyl-L-cysteine (NAC, inhibitor of ROS) was measured by CCK-8 analysis after 48 h post-treatment; (**C**) OC2 and (**D**) OCSL cells were treated with DMSO, piperlongumine in the presence or absence of NAC for 24 and 48 h, and the apoptotic cells were determined with flow cytometry after PI-annexin-V double staining; (**E**) The activation of caspase-3 and PARP-1 was detected under PL in the presence or absence of NAC treatment for 24 and 48 h by Western blotting. Data are presented as the mean ± SD. ** *p* < 0.01; *** *p* <0.001.

Raj *et al.* reported that PL can increase ROS expression and elevate apoptotic cell death in human cancer cells [10]. Therefore, we evaluated the viability of OC2 and OCSL cells under PL treatment and/or NAC treatment for suppressing ROS. Figure 6A,B illustrates the ability of PL to inhibit the survival of PL-treated cells. Cell survival inhibition was significantly reversed by coincubating the cells with NAC (Figure 6A,B), suggesting that ROS were involved in PL-mediated cell death. To confirm that ROS were involved in the PL-induced apoptosis in human OSCC cells, OC2 and OCSL cells were treated with PL in the presence or absence of NAC, and cell apoptosis was determined through flow cytometry. We observed that PL significantly induced apoptosis in both OC2 and OCSL cells, and this phenomenon occurred in a dosage-dependent manner in both cell types (Figure 6C,D). Apoptosis was significantly reduced by cotreating the cells with NAC (Figure 6C,D), suggesting that ROS-related cell apoptosis was involved in PL-mediated cell death. As described previously, PL induced caspase-dependent apoptosis in human OSCC cells (Figure 5B,D). To confirm that ROS were involved in the PL-mediated caspase-dependent apoptosis, the PL-induced expression of caspase-3 and PARP-1 was investigated in the presence or absence of NAC treatment. Figure 6E shows the activation of caspase-3 and PARP-1 after PL treatment; however, it was partially reduced by coincubating the cells with NAC. These results revealed that ROS play a crucial role in PL-induced caspase-dependent apoptosis in human OSCC cells. However, whether PL can elevate ROS accumulation in treated human OSCC cells warrants further investigation.

## 3. Discussion

In this study, we demonstrated the antitumor activity of PL in human OSCCs (Figure 1). Two OSCC cell lines, OC2 and OCSL, established from the buccal specimens of two Taiwanese male patients with a habit of betel quid chewing [19], were used to evaluate the biological function of PL. OC2 cells were more susceptible to PL treatment than were OCSL cells (Figure 1). Recently, PL and its combination with cisplatin in various head and neck cancer (HNC) cells were evaluated by measuring growth, death, cell cycle progression, reactive oxygen species (ROS) production and protein expression, as well as in tumor xenograft mouse models. The results demonstrated PL selectively killed HNC cells, but spared normal cells through the ROS-dependent and JNK/PARP-related death pathway. In addition, PL selectively induced cancer cell death regardless of p53 status [11]. In addition, the IC_50_ levels for OC2 and OCSL in this study are either lower or similar to hepatocellular carcinoma cells (HepG2, HuH7 and LM3) [22], lung cancer cell (A549) [23] and to Krukitt lymphoma cells (DG-75 and Raji) [24] at 24 h after treatment, the IC_50_ levels of which are 6.27 to 20.0 µM. However, the IC_50_ is higher than some Burkitt lymphoma cell lines, such as Daudi (2.8 µM) and Ramos (4.5 µM) [24].

Furthermore, cell cycle arrest at the G0/G1 phase was observed both in OC2 and OCSL cells (Figure 2). The cyclin-dependent kinase inhibitor p21 is a well-known effector of the checkpoint between the G1 and G2 phases of the cell cycle [20]. Moreover, it is involved in various cellular pathways, including cell cycle, senescence and terminal differentiation [21]. Here, we confirmed those observed in a PL-induced cell cycle arrest. PL elevated the expression of p21, suggesting G1 phase arrest (Figure 3). This observation is consistent with that of a previous study on triple-negative breast cancer cells [25]. However, in ovarian cancer cells, PL induces G2/M phase arrest [12], suggesting that cell cycle arrest induced by PL is cell type dependent. Although OCSL cells were more sensitive to PL-induced G1 phase arrest than were OC2 cells (Figure 2), the overall survival rate of OC2 cells was lower than that of the OCSL cells under PL treatment (Figure 1). Moreover, the inhibition of PL-mediated caspase-dependent apoptosis rescued more OC2 cells than OCSL cells (Figure 5D). These data suggest that cell cycle arrest and apoptosis are the major mechanisms involved in PL anti-human OSCC effectivity.

p21 was shown to induce senescence in cancer cells and was considered a checkpoint for limiting the growth of cancer cells [21]. In this study, PL elevated the expression of p21 in human OSCC cells (Figure 3). We further investigated whether PL induced senescence. PL significantly elevated senescence in both OC2 and OCSL cells (Figure 4), suggesting that PL induces p21 expression and senescence in human OSCC cells. We are the first to demonstrate that PL treatment induces senescence in human OSCC cells (Figure 4). Senescence has been recognized as a crucial tumor suppressor mechanism, and senescence-based therapy was identified as a new therapeutic approach [26,27]. However, whether PL-mediated senescence has anti-human OSCC behavior warrants further investigation.

PL has also been reported to induce cellular apoptosis in multiple cancer cells, including ovarian, breast, prostate and Burkett lymphoma cells [10,12,24,28,29]. Here, we observed caspase-dependent apoptosis after PL treatment (Figure 5A–C). This observation is consistent with the findings of previous studies [10,12,24,28,29]. Moreover, OC2 cells were more sensitive to apoptosis than were OCSL cells during PL treatment (Figure 5A), and these findings are consistent with the results in Figure 1A,B and Figure 5B because the cells were treated under the same condition. In addition, OC2 and OCSL cells coincubated with PL and Z-VAD-fmk were significantly resistant to PL-mediated cell death (Figure 5D). However, cell viability reduction was not completely reversed compared to the DMSO-treated groups (Figure 5D), suggesting that PL-mediated caspase-dependent apoptosis plays a key role, but is not the only contributor to anti-human OSCC behavior. In addition, PL-induced autophagy and cell death have been observed in prostate, kidney and breast cancers and osteosarcoma [30,31]. Therefore, whether PL-induced autophagy also plays a role in anti-human OSCC behavior warrants further investigation. Altogether, we demonstrated that multiple mechanisms, including cell cycle arrest, senescence and caspase-dependent apoptosis, contributed to anti-human OSCC activity under PL treatment.

ROS, which are the key mediators of cellular oxidative stress, and redox dysregulation are involved in cancer initiation and progression [13,14,32,33]. ROS and redox dysregulation are observed in multiple cancer cells, and redox dysregulation is a complex phenomenon that integrates many aspects of cancers, including alterations of proliferative control, cancer metabolism and antiapoptotic survival signaling pathways [13,33,34]. Normal cells or non-transformed cells show low basal levels of ROS and express high antioxidant capacity to prevent treatments that impair ROS metabolism [14]. Therefore, it is now widely accepted to constitutively elevate cellular oxidative stress as a promising target for investigating anticancer drugs [14]. PL has been reported to increase ROS selectively in cancer cells rather than in normal cells in multiple cancer cell types, including bladder, colon, breast, pancreatic, lung cancers and glioblastoma [10,35]. PL was also reported to be a potential therapeutic agent for cancer treatment [36]. Our study revealed that PL-mediated anti-human OSCC behavior can be inhibited by NAC treatment, suggesting that ROS play a key role in inhibiting PL-mediated proliferation (Figure 6A,B). Furthermore, the activation of PARP-1 and caspase-3 was suppressed by NAC treatment, and the increase in the level of apoptotic cells was reversed (Figure 6C–E). These data suggest that ROS function upstream from PL-mediated cellular apoptosis. This finding is consistent with the findings previously reported [10,35,37].

## 4. Experimental Section

### 4.1. Cell Lines and Culture

OSCC cell lines, OCSL and OC2, derived from two Taiwanese males with habits of drinking, smoking and betel quid chewing, were maintained in the RPMI 1640 medium supplemented with 10% fetal bovine serum and 1% penicillin/streptomycin. The cells were cultured at 37 °C and supplied with 5% CO_2_.

### 4.2. Cell Viability Assay (CCK-8 Assay)

PL was purchased from Sigma-Aldrich (St Louis, MO, USA). The OC2 and OCSL cells (5 × 10^3^ cells/well) were plated into 96-well cell culture plates and grown in the aforementioned medium. After an overnight attachment period, the cells were treated with the medium alone (containing 0.01% dimethyl sulfoxide (DMSO)) or the medium containing PL. The metabolic activity of the cells was determined using the Cell Counting Kit-8 (CCK-8) assay kit (Sigma-Aldrich). The final results were analyzed using statistical methods in three independent experiments.

### 4.3. Cell Cycle Analysis

The cells (1 × 10^5^) were treated with DMSO or PL after starvation, washed once with phosphate buffered saline and finally fixed using 100% methanol. The fixed cells were stored under airtight conditions at 4 °C. After incubation with RNase (10 mg/mL) and propidium iodide (PI; 1 mg/mL) in the dark for 30 min, the DNA content of the cells was analyzed using FACScan (Becton Dickinson, San Diego, CA, USA) with ModFit LT 3.3 software. Furthermore, the cell cycle marker p21 was determined using Western blotting with an antibody (Epitomics, California, CA, USA).

### 4.4. Apoptotic Cell Death Analysis

To determine PL-mediated apoptosis, cells (1 × 10^6^) were treated with DMSO or PL and were incubated with fluorescein isothiocyanate-labelled annexin V (Sigma-Aldrich) and PI (Sigma-Aldrich) for 15 min at room temperature. The intensity of annexin-V or PI fluorescence was analyzed using FACScan (Becton Dickinson), and 10,000 cells were evaluated in each sample. To confirm the mechanisms underlying PL-mediated apoptosis, the activation of caspase-3 (Cell Signaling; Danvers, MA, USA) and poly(ADP-ribose) polymerase (PARP; Cell Signaling) was detected using Western blotting. In addition, a pan-caspase inhibitor, Z-VAD-fmk (BioVision, Mountain View, CA, USA), was used to reduce caspase-dependent apoptosis, and the cellular viability was determined using CCK-8 analysis. To investigate the role of ROS in PL-mediated apoptosis, the cells were incubated with *N*-acetyl-l-cysteine (NAC; Sigma-Aldrich), an inhibitor of ROS, with or without PL, and the activation of caspase-3 and PARP was detected using Western blotting. Furthermore, cell apoptosis was confirmed through flow cytometry.

### 4.5. DNA Fragmentation Analysis

To confirm that apoptosis was upregulated by PL, DNA fragmentation, which is typically associated with the apoptotic process, was examined. Cells (1 × 10^6^) were plated and treated with DMSO or PL for 48 h, and the genomic DNA was extracted and electrophoretically analyzed on 2% agarose gels containing ethidium bromide (0.1 μg/mL; Sigma-Aldrich).

### 4.6. Senescent Cell Analysis

After treatment with DMSO or PL for 24 h, the cells were washed and fixed with 2% formaldehyde (2%)/glutaraldehyde (0.2%) at room temperature for 5 min and incubated at 37 °C with a fresh senescence-associated β-galactosidase (Sigma-Aldrich) staining solution. The cells were analyzed 72 h after the staining.

### 4.7. Statistical Analysis

Data are presented as the mean ± SD for the indicated number of independent experiments. Differences between the test and control groups were analyzed using one-way ANOVA and the Fisher least significant difference test. Data were statistically evaluated using the Student *t*-test, and the significance was presented as * *p* < 0.05, ** *p* < 0.01 and *** *p* < 0.001.

## 5. Conclusions

In this study, we proved that PL exerts antitumor effects on human OSCC cells through cell cycle arrest and caspase-dependent cellular apoptosis. In addition, ROS were involved in PL-mediated caspase-dependent apoptosis in human OSCC cells. This study is the first to demonstrate PL-induced senescence. Thus, PL is a potential drug for treating human OSCCs and warrants further clinical investigation.

## Supplementary Materials

Supplementary materials can be found at http://www.mdpi.com/1422-0067/17/4/616/s1.
