# Supplementary material for: Piperlongumine Suppresses Proliferation of Human Oral Squamous Cell Carcinoma through Cell Cycle Arrest, Apoptosis and Senescence
Source: Int J Mol Sci. 2016 Apr 23;17(4):616. doi: 10.3390/ijms17040616 (PMC4849064; doi:10.3390/ijms17040616)
Supplement: Supplementary file 1 [file ijms-17-00616-s001.pdf]

Supplementary Materials: Piperlongumine Suppresses Proliferation of Human Oral Squamous Cell Carcinoma through Cell Cycle Arrest, Apoptosis and Senescence

San-Yuan Chen, Geng-Hung Liu, Wen-Ying Chao, Chung-Sheng Shi, Ching-Yen Lin, Yun-Ping Lim, Chieh-Hsiang Lu, Peng-Yeh Lai, Hau-Ren Chen and Ying-Ray Lee

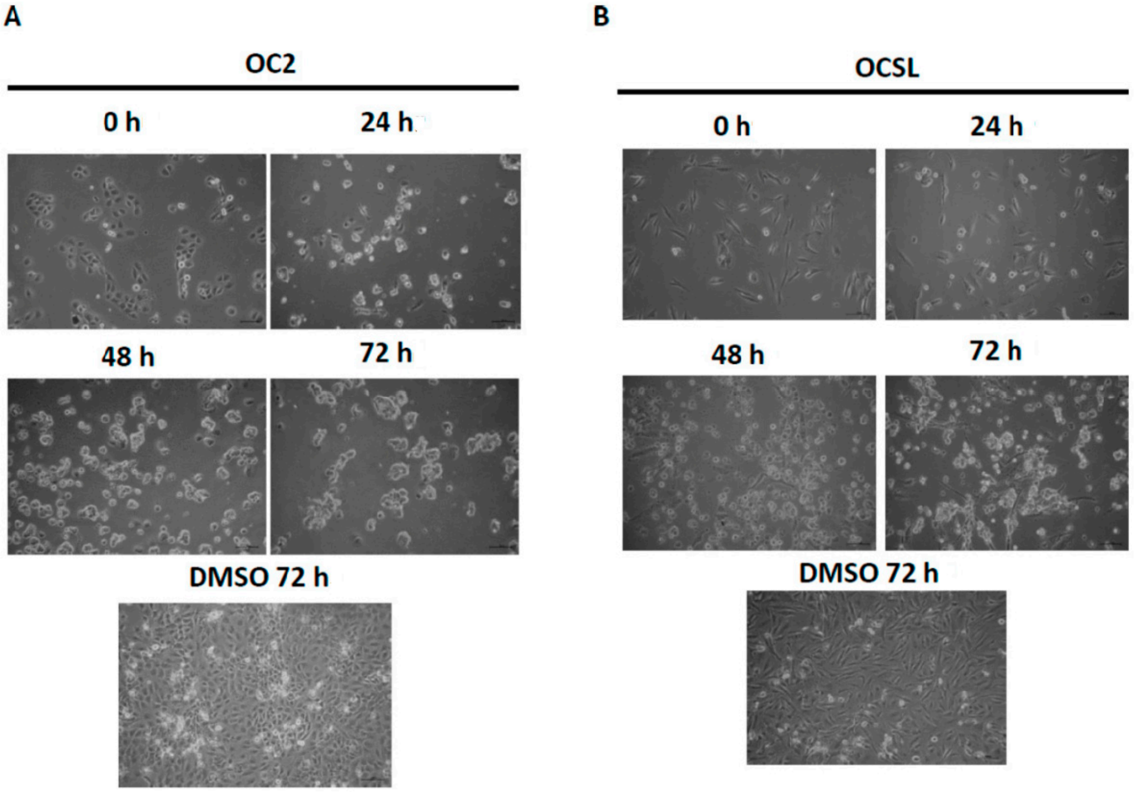

Figure S1. Piperlongumine treatment induced cytotoxicity, which was observed in the significant reduction of the cell number and morphological round-up instead of attachment. OC2 (**A**) and OCSL (**B**) cells were incubated with DMSO and piperlongumine (10 μM) for various times, and the morphologic changes were observed under a microscope.

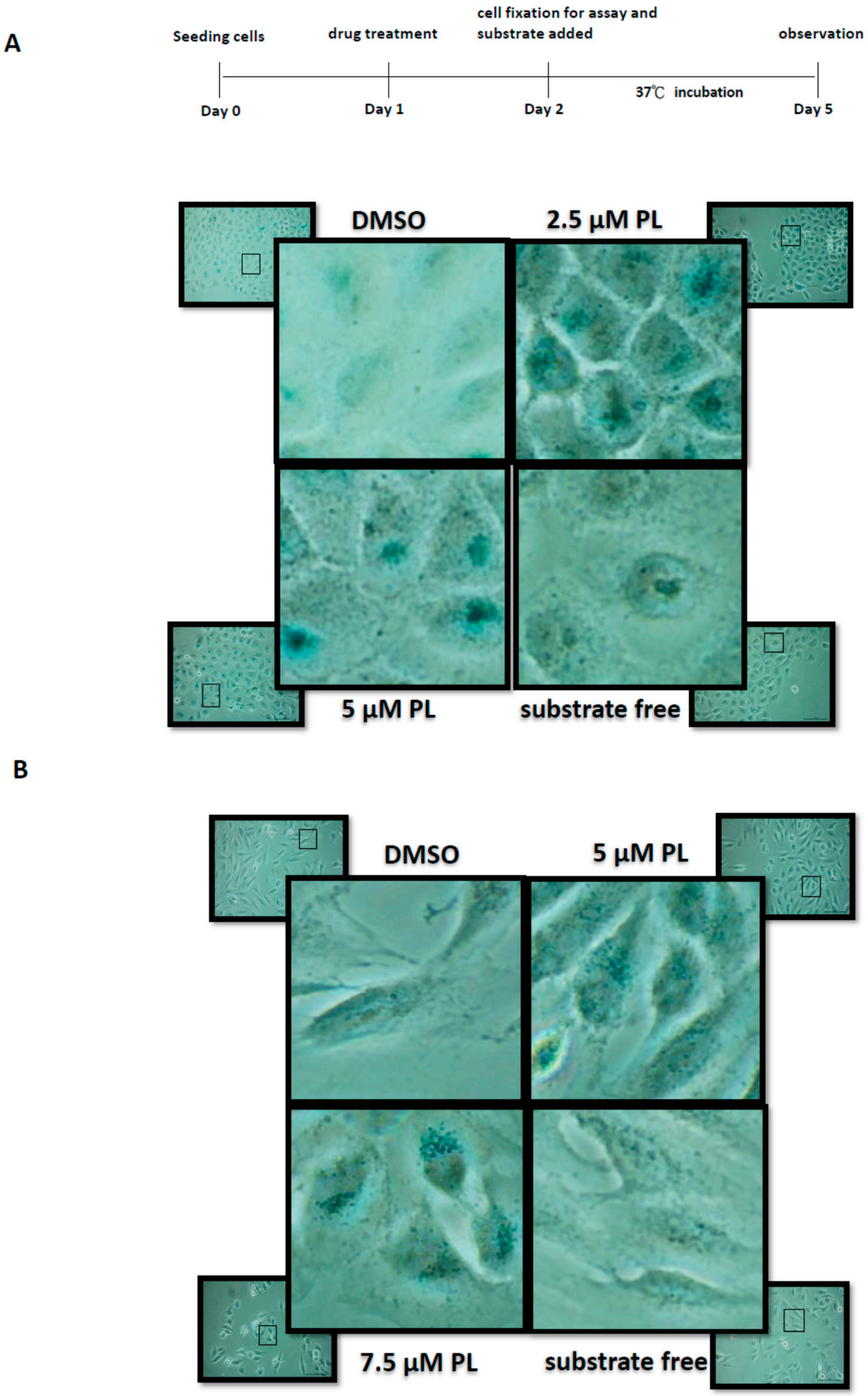

Figure S2. Piperlongumine induced cellular senescence in human OSCC cells. After treatment with DMSO or piperlongumine for 24 h, (**A**) OC2 and (**B**) OCSL cells were stained with SA-β-Gal, and the senescent cells were determined under a microscope.
